# Effects of selective cyclooxygenase‐2 inhibitor robenacoxib on primary cells derived from feline injection‐site sarcoma

**DOI:** 10.1111/jcmm.17717

**Published:** 2023-06-19

**Authors:** Chen‐Hui Lu, Shu‐Han Yu, Ching‐Ho Wu, Jason Lih‐Seng Yeh, Hui‐Wen Chang, Chian‐Ren Jeng, Yen‐Chen Chang

**Affiliations:** ^1^ School of Veterinary Medicine, Graduate Institute of Molecular and Comparative Pathobiology National Taiwan University Taipei Taiwan; ^2^ Institute of Biotechnology National Taiwan University Taipei Taiwan; ^3^ School of Veterinary Medicine, Institute of Veterinary Clinical Science National Taiwan University Taipei Taiwan

**Keywords:** cyclooxygenase‐2, feline injection‐site sarcoma, primary cells, robenacoxib

## Abstract

Feline injection‐site sarcomas (FISSs) are highly invasive malignant mesenchymal neoplasms that arise from injection sites in cats. Although the tumorigenesis of FISSs is still uncertain, there is a consensus that FISS is associated with chronic inflammation caused by irritation of injection‐related trauma and foreign chemical substances. Chronic inflammation can provide a proper microenvironment for tumour development, which has been known as one of the risk factors of tumorigenesis in many tumours. To investigate the tumorigenesis of FISS and screen for its potential therapeutic targets, cyclooxygenase‐2 (COX‐2), an inflammation‐enhancing enzyme, was selected as a target for this study. In vitro experiments using FISS‐ and normal tissue‐derived primary cells and robenacoxib, a highly selective COX‐2 inhibitor, were performed. The results demonstrated that expression of COX‐2 could be detected in formalin‐fixed and paraffin‐embedded FISS tissues and FISS‐derived primary cells. Cell viability, migration and colony formation of FISS‐derived primary cells were inhibited, and cell apoptosis was enhanced by robenacoxib in a dose‐dependent manner. However, susceptibility to robenacoxib varied in different lines of FISS primary cells and was not completely correlated with COX‐2 expression. Our results suggest that COX‐2 inhibitors could be potential adjuvant therapeutics against FISSs.

## 1 INTRODUCTION

Feline injection‐site sarcoma (FISS) is a unique neoplastic disease in cats that was first reported in 1991.^1^ Feline injection‐site sarcomas are a group of malignant mesenchymal tumours and are considered to be correlated with injective events,^2^ including vaccination,^1^, ^3^, ^4^ drug administration,^5^, ^6^, ^7^ and implantation of microchips.^8^, ^9^ Under histopathological examination, FISSs are characterized by aggregation of inflammatory cells at the periphery of neoplastic growth and sometimes with foreign bodies, such as aluminium‐based adjuvant or non‐absorbable surgical material.^10^, ^11^ Compared with sarcomas not associated with injection, FISSs tend to have more prominent malignancies and aggressive,^4^, ^12^, ^13^ but with relatively low metastatic rates, ranging between 0%–28%.^14^ The prognosis of FISSs is mainly dependent on local recurrence but usually poor. Surgical excision with wide margins is the preferred therapy for FISSs. However, owing to the high local invasiveness of FISSs, single excisional surgery or radiotherapy is commonly not curative.^14^, ^15^ Thus, radiotherapy is the current mainstream adjuvant therapy combined with surgery.^15^ Other types of treatments, such as chemotherapy and immunotherapy are rarely administered clinically because of their uncertain therapeutic efficiency.

The pathogenesis of FISSs remains unclear. Currently, the most widely accepted consensus is that FISSs might arise from chronic inflammation of injected sites due to evidence of the presence of aluminium, peripheral multinucleated giant cells,^4^, ^16^ peripheral aggregates of inflammatory cells, and a significant epidemiological association between vaccination and sarcomas. Malignant transformation might be induced by excessive inflammatory and immunological responses, which could be stimulated by the adjuvant or other components of the vaccine, followed by abnormal proliferation of fibroblasts and myofibroblasts.^4^, ^17^ Chronic inflammation should be regarded as a tumour promoter and carcinogen,^18^ such as in human colorectal cancer with intestinal bowel disease,^19^ human hepatocellular carcinomas with hepatitis B or C viral infection,^20^ and ocular sarcoma with trauma and chronic uveitis in cats.^17^ Chronic inflammation involves various immunocytes and factors contributing to the chemotaxis of immunocytes, remodelling of extracellular matrix, removal of pathogens and healing of damaged tissue.^21^ Sustained and uncontrolled inflammation leads to a consistent cycle of tissue damage, proliferation and healing^22^; triggers mutations resulting in neoplastic transformation, including silencing of tumour suppressor genes and activation of oncogenes^23^; and develops an adequate microenvironment beneficial to oncogenesis, tumour proliferation, invasion and metastasis.^20^, ^22^

Cyclooxygenase‐2 (COX‐2) is an isoform of cyclooxygenase that converts arachidonic acid into prostaglandins, which are known as inflammatory promoters and are considered to collaboratively contribute not only to chronic inflammation but also to an adequate microenvironment for tumorigenesis.^24^, ^25^ Additionally, overexpression of COX‐2 has been reported in various human tumours,^26^ canine^27^, ^28^, ^29^ and feline,^29^, ^30^, ^31^, ^32^, ^33^ including FISSs.^34^, ^35^, ^36^ In human medicine, it has been reported that COX‐2 inhibitors could sensitize tumour cells to enhance their response to radiotherapy or chemotherapy. It has also been suggested that chemotherapy drugs or radiotherapy adjuvants with COX‐2 inhibitors may provide favourable synergistic responses to antitumor activity.^25^ Additionally, previous work showed that nuclear factor kappa B (NF‐κB), which is upstream of COX‐2, may play a role in FISS tumorigenesis.^37^ Accordingly, COX‐2 may be a potential therapeutic target for oncogenesis in veterinary medicine. Robenacoxib is a novel COX‐2 selective inhibitor with sufficient safety in cats and more intense COX‐2 selectivity than meloxicam.^38^, ^39^ However, only a few studies have used COX inhibitors for feline tumour treatments,^40^, ^41^ and the effect of robenacoxib or COX inhibitors on FISSs has not yet been investigated. This study aimed to investigate the role of COX‐2 in the oncogenesis of FISS and to evaluate the therapeutic potential of the selective COX‐2 inhibitor, robenacoxib, in FISS‐derived primary cells with respect to proliferation, migration, apoptosis and clonogenic ability.

## 2 METHODS

### 2.1 Cells

Feline injection‐site sarcoma primary cell lines were established as previously described,^37^ and the FISS cell lines used in the present study were FISS‐7 and FISS‐12. To obtain normal feline mesenchymal (FM) cells, a normal muscle tissue was collected from a cat euthanized due to other diseases. The culture and passage of FM cells were similar to those of previously described FISS cell lines.

### 2.2 Immunohistochemistry staining (IHC)

For immunohistochemical staining, 10 micrometre‐thick slices were prepared from formalin‐fixed and paraffin‐embedded (FFPE) tissue blocks of FISSs and normal canine kidneys.^35^ The slides were first deparaffinized, rehydrated and then immersed in Trilogy (Cell Marque). Antigen retrieval was conducted by two rounds of heating at 95°C for 5 min using EZ‐Retriver System (BioGenex Laboratories Inc.). After washing with Tris‐buffered saline supplemented with 0.1% Tween® 20 (TBST, Genestar Biotech Inc.), the tissue slides were blocked with 10% of normal goat serum (Dako) diluted in phosphate‐buffered saline (PBS) at room temperature (RT) for 30 min and followed with another TBST washing. The COX‐2 polyclonal antibody (Cayman Chemical)^35^ was diluted 1:200 with PBS and applied to the slides at RT for 1 h. Following the TBST washing, incubation was performed in 3% hydrogen peroxide for 15 min at RT to block the endogenous peroxidase. EnVision® + Dual Link System‐HRP (DAB+) (Dako) was used for development according to the manufacturer's protocol. Next, the slides were counterstained with haematoxylin for 20 s and then washed for at least 5 min with running tap water. Finally, after being air‐dried and sealed with gum arabic and coverslips, the slides were evaluated using an optical microscope by two pathologists. To quantify the positive cells in the tumour, ten high‐power fields were randomly selected and captured, and the pictures were analysed by using ImageJ software (NIH). The ratio was calculated by dividing the positive cell count by the total cell count.

### 2.3 Immunocytochemistry staining (ICC)

To identify the origin of primary FM cells obtained from muscular tissue and FISS‐derived cells, ICCs were incubated with vimentin, desmin and anti‐alpha‐smooth muscle actin (α‐SMA) antibodies. The cells were seeded onto a 96‐well plate (4.5 × 10^3^ cells/well) and incubated at 37°C for 24 h to reach 80%–90% confluence. After removal of the culture medium, the cells in each well were fixed with 80% acetone at −20°C for 10 min, air‐dried at RT and stored at −20°C for subsequent use. One hundred microliters of anti‐vimentin antibody (1:800 dilution) (Dako), anti‐desmin antibody (1:200 dilution) (Dako) and α‐SMA antibody (1:800 dilution) (Dako) were added to each well and incubated for 1 h at RT. After washing with PBS, the EnVision® + Dual Link System‐HRP (DAB+) (Dako) was used following the manufacturer's protocol. The images were evaluated using an inverted microscope (Eclipse TS 100; Nikon) by two pathologists. To quantify the positive cells in the tumour, five high‐power fields were randomly selected and captured and these pictures were analysed using ImageJ software (NIH). The ratio was calculated by dividing the positive cell count by the total cell count. To detect the expression of COX‐2 in FISS and FM cells, ICC was conducted as previously described. The primary antibody was replaced with a COX‐2 polyclonal antibody (Cayman) diluted at 1:200 in PBS.

### 2.4 Immunoblotting to detect the expression of COX‐2 in FISS and FM cells

Western blotting was performed to evaluate the expression levels of COX‐2 in FISS‐7, FISS‐12 and FM cells. Semi‐quantification of COX‐2 was conducted by measuring the fold changes in COX‐2 compared to β‐actin, as a housekeeping control. To prepare cell lysates, 1 mL of RIPA lysis buffer (VWR Chemicals, Sohn) was added to the cells in a 75 T sterile cell culture flask (Thermo Fisher Scientific) with full confluence. The cell lysate of each cell was mixed with lithium dodecylsulfate loading buffer (Invitrogen, Thermo Fisher Scientific) and reducing buffer (Invitrogen) according to the manufacturer's protocol and was heated at 95°C for 5 min. The samples were separated by 10% sodium dodecyl sulfate‐polyacrylamide electrophoresis gel and transferred to polyvinylidene difluoride (PVDF) membrane (Bio‐Rad, Hercules). Next, the PVDF membrane was blocked with 5% skim milk in TBST (Genestar Biotech Inc.) and subsequently washed with TBST buffer. The blot was probed with β‐actin antibody (1:2000 dilution in TBST buffer, GeneTex) or anti‐COX‐2 (1:200 dilution in TBST, Cayman) (Figure S2) and then washed with TBST buffer. Next, the blot was incubated with horseradish peroxidase‐conjugated secondary anti‐rabbit antibody (1:10000 dilution in blocking buffer; Jackson ImmunoResearch Laboratories), followed by washing with TBST. Protein development was performed using Clarity™ Western ECL Blotting Substrates (Bio‐Rad) and ChemiDocTM Imaging Systems (Bio‐Rad).

### 2.5 Reagents

The highly selective COX‐2 inhibitor, 5‐Ethyl‐2‐[(2,3,5,6‐tetrafluorophenyl) amino] benzeneacetic acid (robenacoxib; BioVision Inc.) was dissolved in dimethyl sulfoxide (DMSO; Mallinckrodt Chemical Works) at a final concentration of 0.2 M stock solution and stored at −20°C for use.

### 2.6 AlamarBlue cell viability assay for cell viability analysis

The FISS or FM primary cells seeded onto 96‐well plates (NUNC, Thermo Fisher Scientific) at a number of 4.5 × 10^3^ cells per well were incubated at 37°C for 24 h and treated with culture medium supplemented with robenacoxib at concentrations of 125, 250, 500 and 1000 μM, respectively. The cells in the negative control group were incubated in a culture medium supplemented with 0.5% DMSO. After the incubation for 24 and 72 h, the AlamarBlue Cell Viability Assay Reagent (G‐Biosciences) was used to assess the inhibitory effects of robenacoxib according to the manufacturer's protocol. The 96‐well plate was read using the Cytation 7 Cell Imaging Multi‐Mode Reader (BioTek Instruments) at 570 and 600 nm, respectively. The percentage reduction was calculated using the protocol provided. The half‐maximal inhibitory concentration (IC_50_) of robenacoxib was calculated by normalized inhibitory response nonlinear regression analysis using PRISM 9 (GraphPad Software).

### 2.7 Wound healing assay to evaluate the ability of cell migration

Approximately 2 × 10^5^ FISS or FM cells per well were seeded in a 24 well‐plate (NUNC, Thermo Fisher Scientific) and incubated at 37°C for 24 h to reach 90%–100% confluence. Wounds were generated by scratching the cell monolayers with a pipette tip. Subsequently, the medium was replaced with culture medium supplemented with 125, 250, 500 or 1000 μM of robenacoxib or 0.5% DMSO, respectively. Each treatment was conducted in triplicates. Photographs were taken at 0 and 24 h, and the width of the wounds was measured using the ImageJ software (NIH). Because there were still several remaining cells after the scratch at 0 h, the edge of cell migration was defined as where the cells reached at least 50% confluence. Ten measurements at different sites were performed to obtain the average widths of the wounds at each treatment and time point. The data are presented as a percentage compared to the average value at 0 h for each treatment.

### 2.8 Clonogenic assay for cell colonization

A clonogenic assay was used to evaluate the ability of cells to colonize after drug treatment. To obtain a single cell suspension, the primary cells were digested using StemPro™ Accutase™ Cell Dissociation Reagent, suspended in DMEM supplemented with 125, 250, 500 or 1000 μM robenacoxib, and passed through a sterile 40 μm cell strainer (Fisherbrand, Waltham, Massachusetts, United States). Thereafter, these cells at a number of 10^3^ cells/well were seeded onto the six‐well plates (NUNC, Thermo Fisher Scientific). Cells treated with culture medium added with 0.5% DMSO were designated as the vehicle control. All treatments were conducted in triplicates. After a three‐day incubation period, the medium was removed and replaced with a drug‐free culture medium every 3–5 days until cell colonies could be detected by the naked eye (more than 30 cells in each colony). To determine the number and size of cell colonies, the cells were washed with cold PBS (Gibco) and stained with 0.1% crystal violet solution in 20% ethanol at RT for 10 min. The results were evaluated by plating efficiency (PE) and survival fraction (SF).^42^ Plating efficiency was defined as the percentage of colony formation and number of cells seeded in the well, and SF was defined as the number of colonies formed after treatment divided by the number of cells seeded in multiple PE.

### 2.9 Terminal deoxynucleotidyl transferase dUTP nick end labelling (TUNEL) assay for cell apoptosis

To evaluate the effect of robenacoxib on apoptosis, the DeadEnd™ Fluorometric TUNEL system (Promega, Madison) was used. Approximately 4.5 × 10^4^ cells per well were seeded in the 24‐well plates (NUNC, Thermo Fisher Scientific) and incubated at 37°C overnight. Next, the culture medium in each well was replaced with a culture medium containing 125, 250, 500, or 1000 μM of robenacoxib or 0.5% DMSO for one‐day incubation. All treatments were conducted in triplicates. The treated cells were suspended and then deposited on slides using Shandon CytoSpin 3 (Thermo Fisher Scientific Shandon) for subsequent TUNEL staining. The cells were fixed and stained following the protocol. The slides were read and analysed under a fluorescence microscope (IX83; Olympus). Under 200X magnification, eight randomly selected areas of each slide were photographed and counted using ImageJ (NIH).

### 2.10 Statistical analysis

Statistical analysis was performed using SAS version 9.4 (SAS Institute, Cary, North Carolina, United States). Group comparisons between the controls and treatments were conducted using a one‐way analysis of variance with the Scheffé test. Statistical significance was defined as a *p*‐value <0.05. The IC_50_ and its inhibitory curves were analysed using Prism 9 (GraphPad Software).

## 3 RESULTS

### 3.1 Immunophenotypes in primary cells derived from FISS and normal feline tissues via ICC staining

**FIGURE 1:**
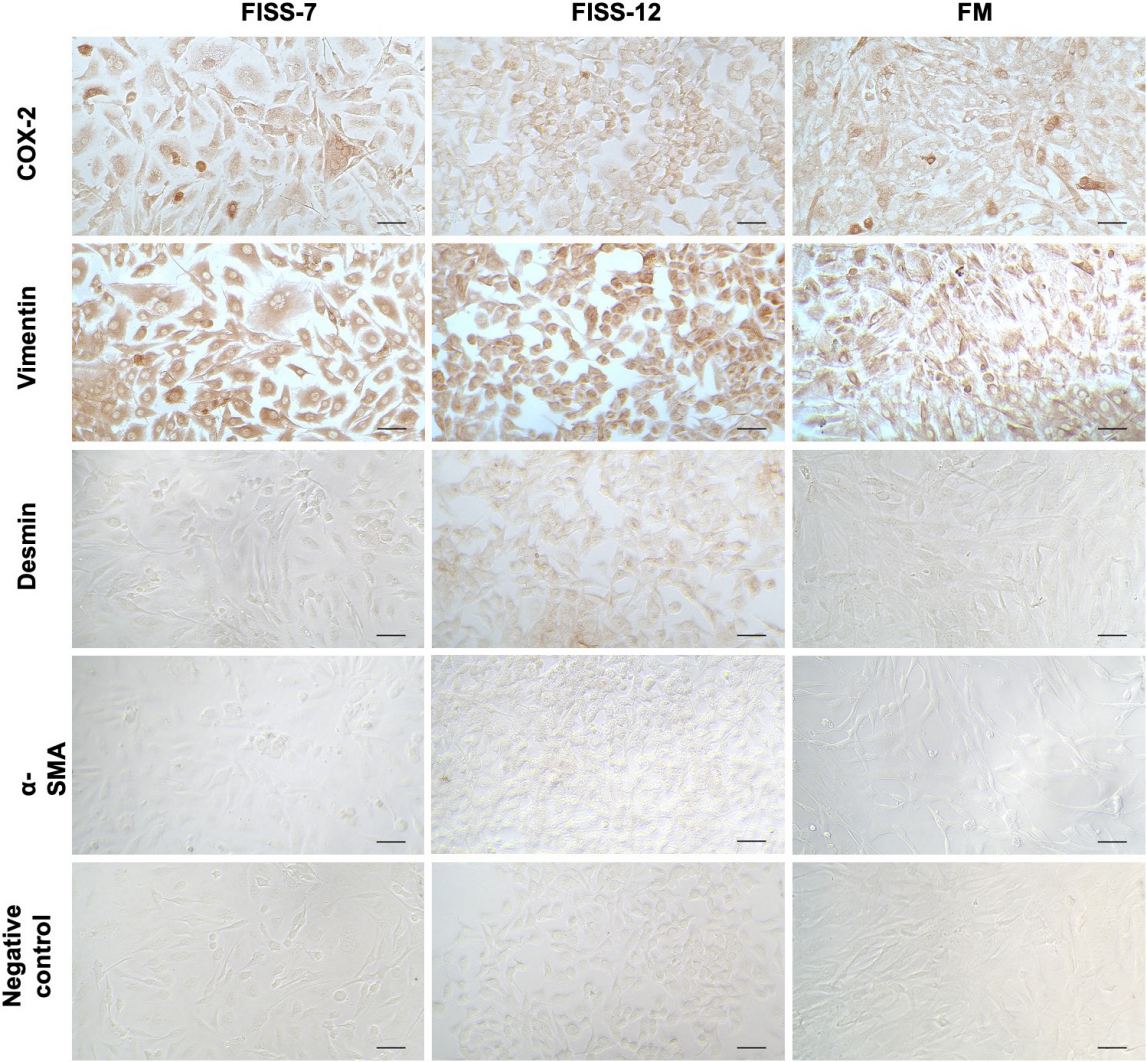
Expression level of COX‐2, vimentin, desmin and α‐SMA in FISS‐7, FISS‐12 and FM primary cells. Negative controls are stained with phosphate‐buffered saline as primary antibody. Length of the scale bars is 50 μm.

Three isolates of primary cells were used in the present study, including cells derived from normal feline tissues and FISSs. Histopathologically, the original masses of both FISS cells were categorized as grade III soft tissue sarcomas according to the grading system published by Dennis et al.^43^; the detailed information is listed in Table S1. Immunocytochemical staining for vimentin, desmin and α‐SMA was performed and the results are shown in Figure 1. All the primary cells displayed strong diffuse immunoreactivity to vimentin. FISS‐12 cells exhibited diffuse and weak desmin immunoreactivity, but FISS‐7 cells and cells from normal tissue were negative for desmin. All primary cells were negative for α‐SMA expression. The results indicated that FISS‐7 cells and normal tissue‐derived primary cells were fibroblasts of origin and FISS‐12 cells were skeletal muscle in origin. The primary cells derived from normal feline tissue are named feline mesenchymal cells (FM) in the following text.

### 3.2 COX‐2 expression in FISS‐7 and FISS‐12

**FIGURE 2:**
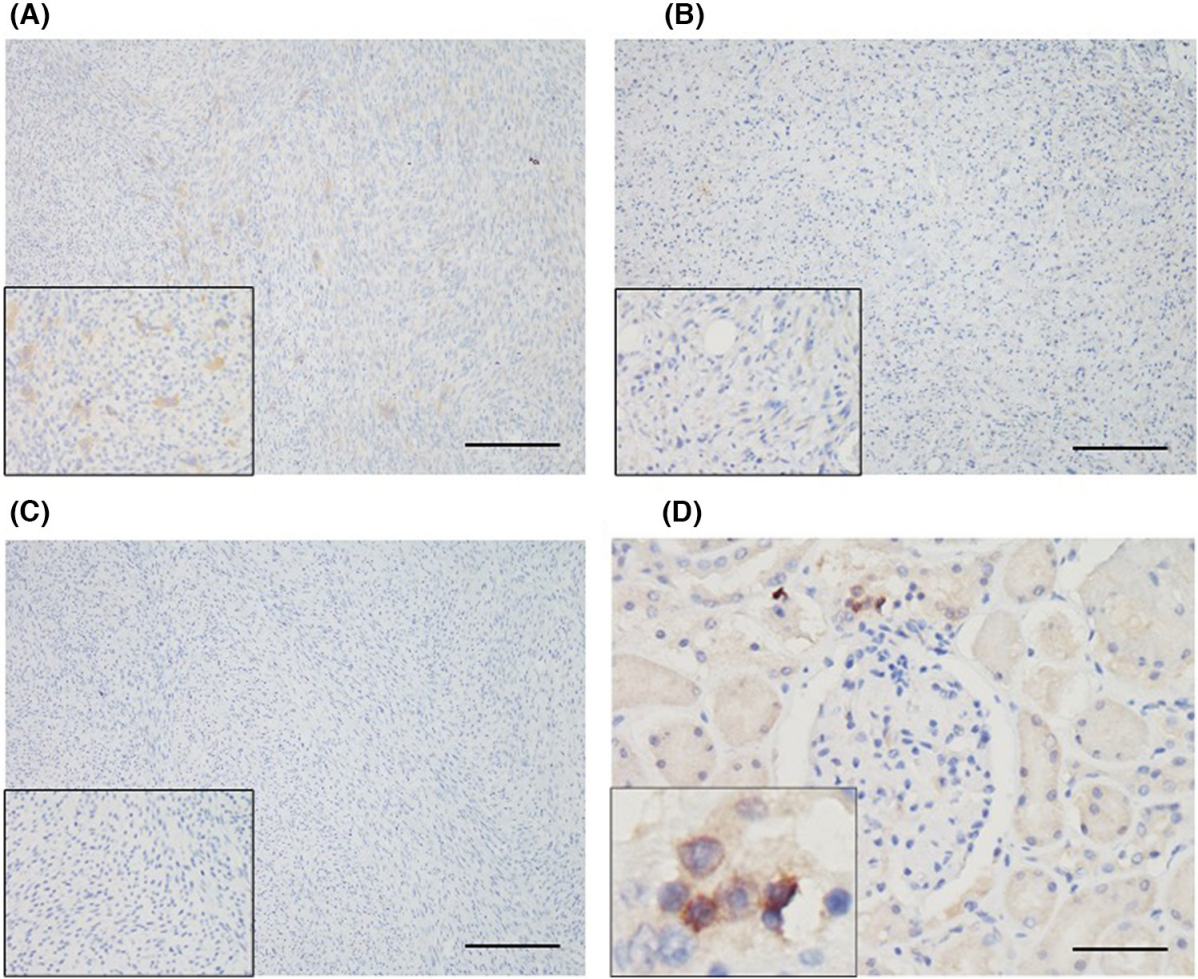
Cyclooxygenase‐2 expression in feline injection site sarcomas (FISSs) by immunohistochemical staining. Results of FISS‐7 and FISS‐12 are displayed in (A) and (B), respectively. Insets showing higher magnifications of positive cells (400×). (C) Negative control was stained with phosphate‐buffered saline and the result is shown. (D) The macula densa in normal canine kidney is exhibited as positive control. The scale bars showed in (A–C) and (D) are 200 and 50 μm, respectively.

**FIGURE 3:**
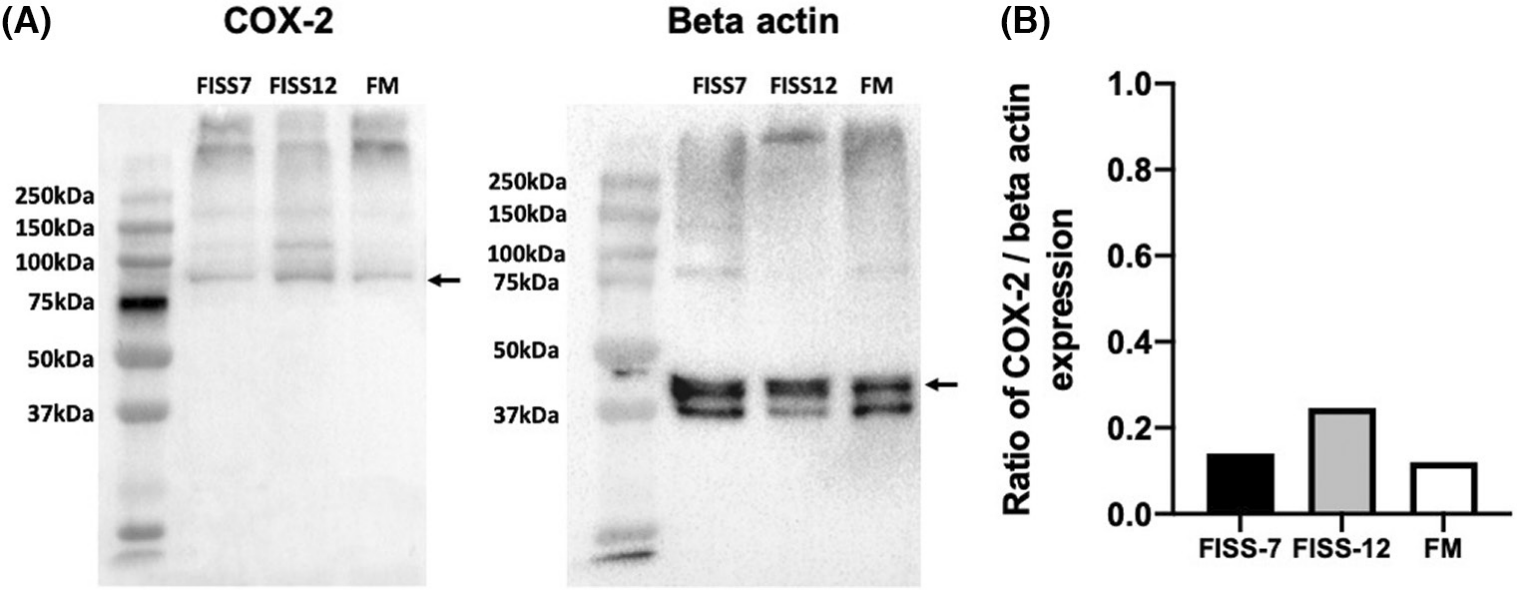
Result of western blotting against COX‐2 and beta Actin antibody in FISS and FM cells. (A) Back arrows indicate respective target proteins. (B) The quantified COX‐2 expression levels in different primary cells. The ratio of each primary cell is the fold change of COX‐2 expression when compared to beta Actin expression. No statistical difference is shown between the cell lines.

To evaluate the expression of COX‐2 in FISS cells, ICC and IHC staining and western blotting against COX‐2 were performed (Figure 1). In ICC staining, FISS‐7 cells displayed diffuse COX‐2 immunoreactivity and the multinucleated cells (<5%) had strong intracytoplasmic signals. The expression pattern of the FM cells was similar to that of FISS‐7 that there were a small number of cells (<5%) demonstrating strong cytoplasmic positivity. Compared to FISS‐7 and FM cells, FISS‐12 cells were diffusely positive for COX‐2 without strong positive staining. For IHC staining, the macular densa of the normal canine kidney was used as the positive control for COX‐2 staining. The results are shown in Figure 2. COX‐2 expression was observed in the cytoplasm of the macular densa of the canine kidney. In FISS‐7, approximately 5% of the tissue showed mild to moderate immunoreactivity, especially in the multinucleated giant cells. In contrast to FISS‐7, FISS‐12 tissues had <1% positive cells. Generally, the results of IHC staining are much weaker than those of ICC staining. Western blotting (Figure 3A) revealed the expression of β‐actin in FISS‐7, FISS‐12 and FM cells. The blot stained with anti‐COX‐2 antibody showed protein bands between 75 kDa and 100 kDa. The fold‐change of COX‐2 compared to β‐actin was measured and calculated, and the results are shown in Figure 3B. This shows that FISS‐12 cells had the highest COX‐2 expression level among the three primary cell lines.

### 3.3 Safety of COX‐2 inhibitor robenacoxib in FISS and FM cells

To investigate the role of COX‐2 in the pathogenesis of FISSs, the effect of robenacoxib on FISS cells was studied to investigate the present study. Drug safety was evaluated by performing the AlamarBlue cell viability assay and presented as IC_50_. The IC_50_ values of each primary cell line after 24 and 72 h of treatment are shown in Figure S1 and Table 1. Comparing with FISS‐12 (734.0 μM) and FM cells (773.9 μM), the IC_50_ of FISS‐7 was 488.5 μM, indicating that robenacoxib had higher toxicity and survival inhibition than FISS‐7. The cell viability values at different concentrations of robenacoxib at 24 and 72 h are presented in Table S2–S4. The viability of all three primary cells decreased in a dose‐dependent manner, and a significant decrease in cell survival rate was observed at the highest concentration (1000 μM) and was also observed at 500 μM in FISS‐7. However, there was no obvious difference in the IC_50_ and cell viability between FISS‐12 and FM cells.

**TABLE 1:** The half maximal inhibitory concentration (IC_50_) of robenacoxib in FISS and FM primary cells at 24 and 72 h of incubation.

Cells	24 h	72 h
FISS‐7	472.6 μM	488.5 μM
FISS‐12	1014.0 μM	734.0 μM
FM	972.8 μM	773.9 μM

### 3.4 Cell migration effects of robenacoxib on FISS and FM cells, as measured by a wound healing assay

**FIGURE 4:**
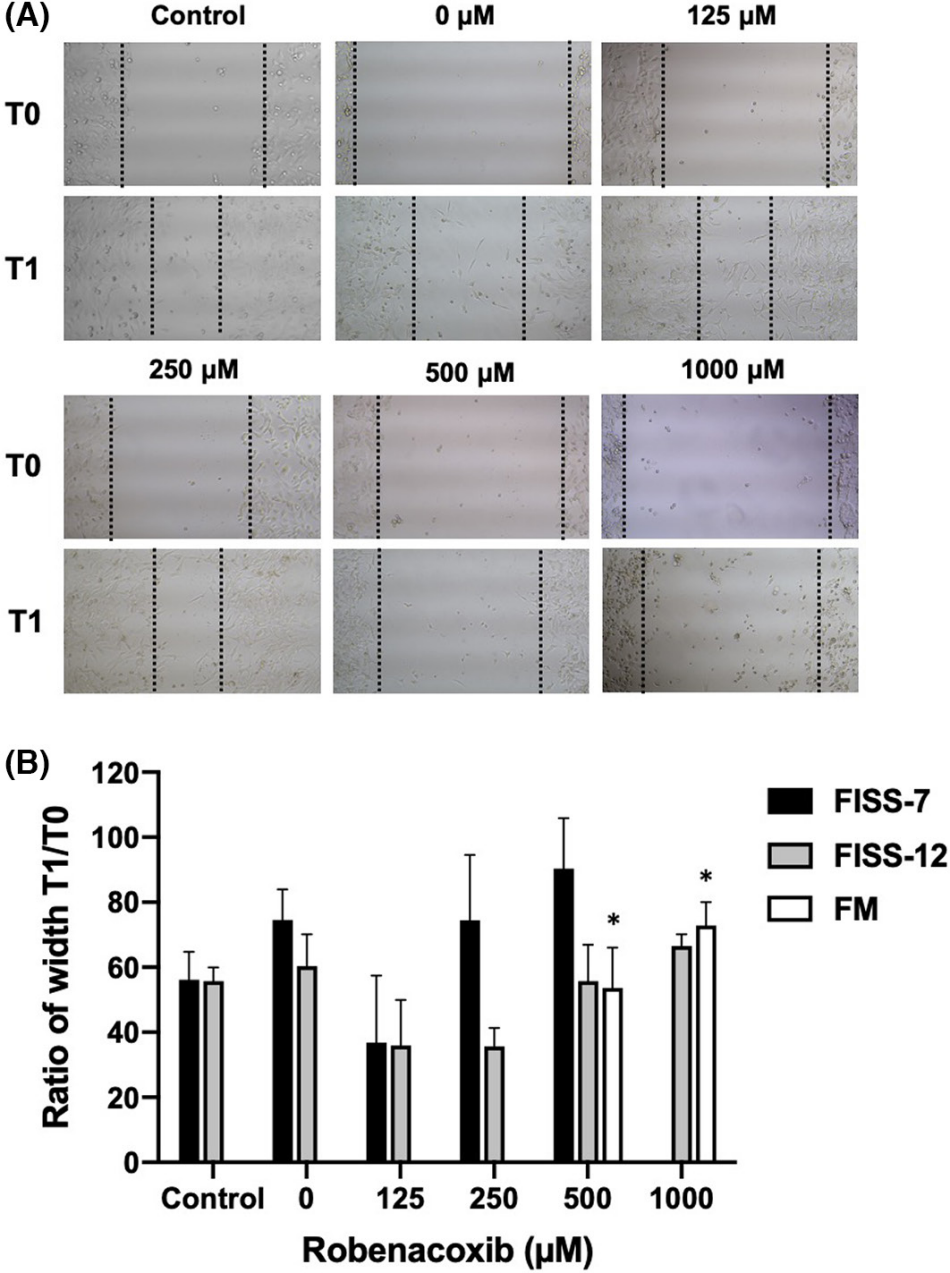
Results of wound healing assay in FISS and FM cells under different concentrations of robenacoxib. (A) Figures illustrate cell migration of FISS‐12 at different concentrations and time points. Widths of the wounds were photographed and measured before (T0) and 24 h (T1) after being treated with different concentrations of robenacoxib. Treatment with 0 μM robenacoxib is considered as a vehicle (DMSO) control, in which the culture medium is only supplemented with 0.5% DMSO. (B) Values of T1 are expressed as the percentages of the wound widths at T0. * Values are statistically significant when compared to the medium control (*p* < 0.05).

A wound healing assay was performed to evaluate the effect of robenacoxib on the migration of different primary cells, and the results are shown in Figure 4 and Table S5–S7. Robenacoxib reduced the migration ability of FISS‐7 and FISS‐12 cells. However, compatible with the results of cell viability, FISS‐7 cells treated with 1000 μM robenacoxib were almost completely exfoliated, leading to unrecognizable scratches. In contrast, compared with FISS‐7 and FISS‐12, the distance of the wounds in FM cells treated with 500 and 1000 μM was apparently narrowed (*p* < 0.05), but the wounds at 0, 125 and 250 μM and the medium control healed completely after 24 h. Briefly, the inhibitory effect of robenacoxib on cell migration varied in different lines of primary cell types and at different concentrations of the drug.

### 3.5 Effect of robenacoxib on the cell clonogenesis in FISS and FM cells

**FIGURE 5:**
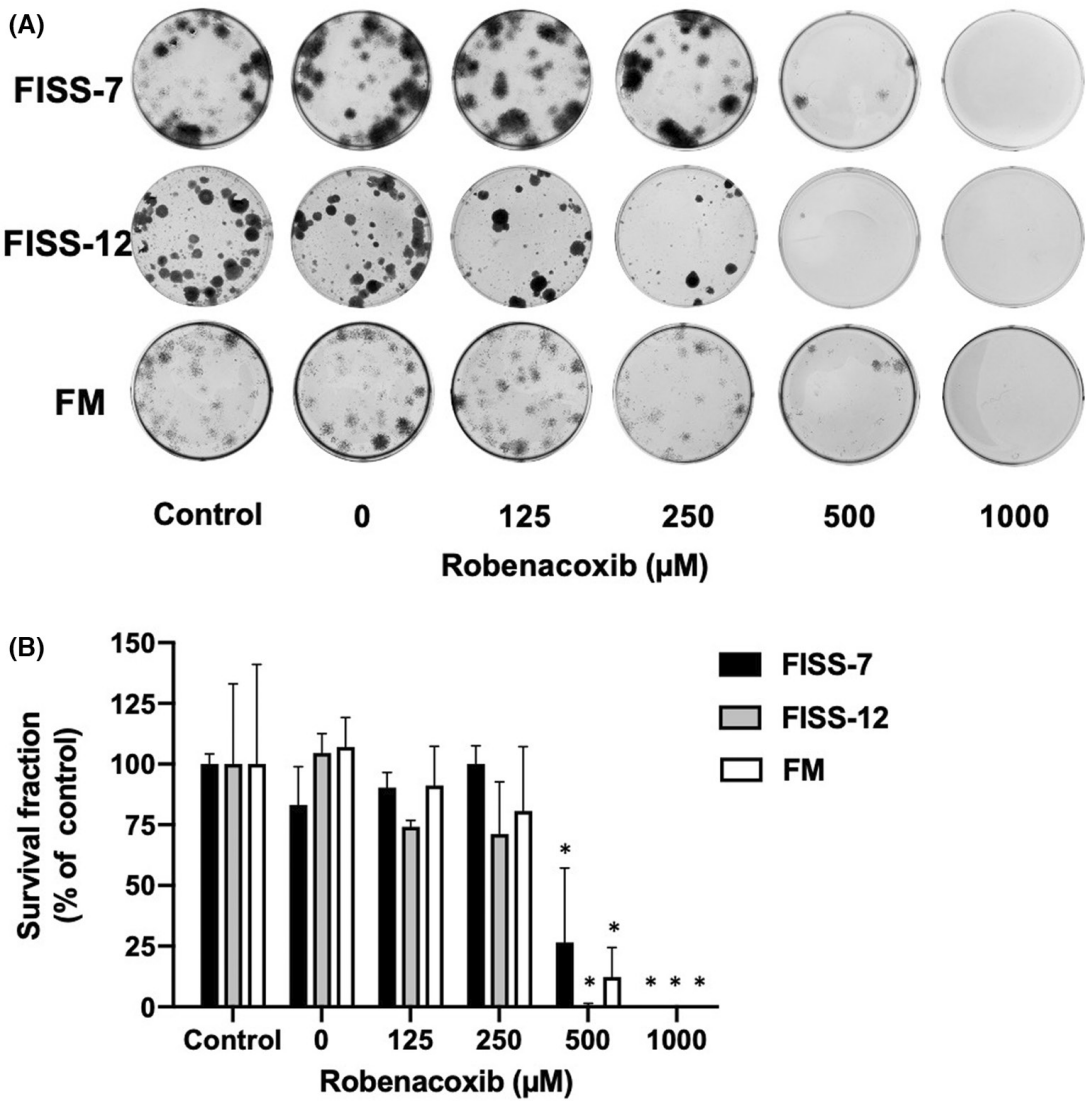
Results of clonogenic assay in different lines of primary cells treated with different concentrations of robenacoxib. (A) Graphs of the cell clonogenesis of each line of primary cells on the six‐well plate. (B) Survival fraction of each primary cell line treated with different concentrations of robenacoxib are illustrated. Group treated with 0 μM robenacoxib is designated as vehicle (DMSO) control group. Group treated with medium only is designated as control group. * Represents significant statistical difference than the control group (*p* < 0.05).

To study the effect of robenacoxib on cell colony formation, clonogenic assays of FISS‐7, FISS‐12 and FM cells were performed, and the results are displayed in Figure 5 and Table S8. The results showed that all lines of primary cells shared a similar pattern with higher concentrations of robenacoxib, including 500 and 1000 μM, exhibiting a significant inhibitory effect on colony formation (*p* < 0.05). As for cells treated with 0, 125, or 250 μM robenacoxib, there was no significant difference in each cell when compared to that of their own medium control (*p* > 0.05). At 500 μM, FISS‐7 and FM cells tended to have more colonies than FISS‐12 cells, suggesting that the clonogenesis of FISS‐12 may be more sensitive to robenacoxib than FISS‐7 and FM cells.

### 3.6 Apoptosis rate of FISS and FM cells detected using TUNEL assay

**FIGURE 6:**
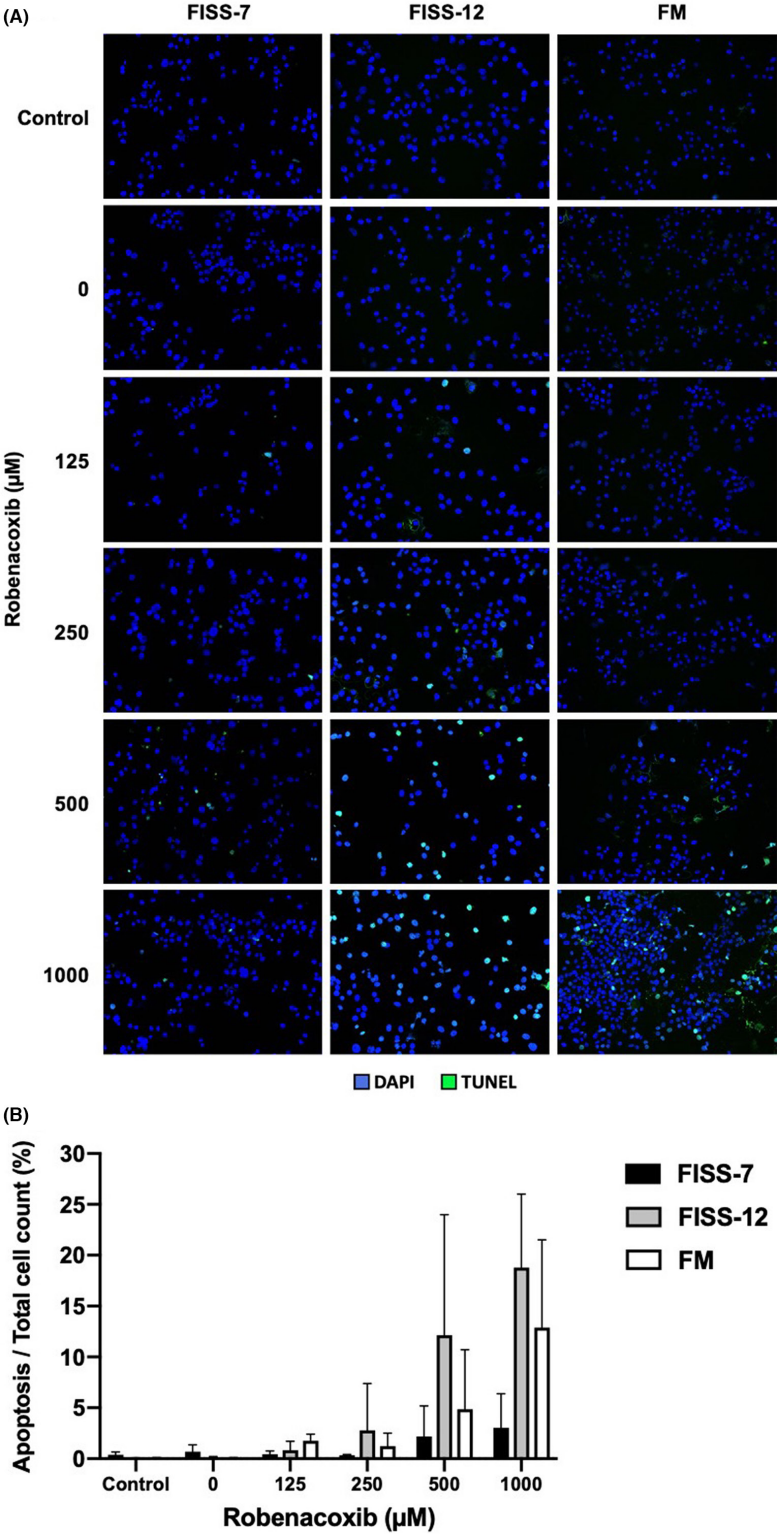
Result of TUNEL assay for evaluating cell apoptosis in different lines of primary cells treated with different concentrations of robenacoxib. (A) Apoptotic cells detected by TUNEL assay in each line of primary cells after treatment with different concentrations of robenacoxib. Green spots are positive signals of TUNEL staining. Blue spots are the cell nuclei stained with DAPI as counterstain. Double‐positive cells of TUNEL and DAPI staining highlighted as light blue are interpreted as the apoptotic cells. (B) Result of apoptotic rate expressed as percentage of apoptotic cell count to total cell count. Group treated with 0 μM of robenacoxib is designated as the vehicle (DMSO) control group. Group treated with medium only is designated as the control group. * Represents significant statistical difference than the control group (*p* < 0.05).

To evaluate the effects of robenacoxib on apoptosis in FISS‐7, FISS‐12 and FM cells, TUNEL assay was performed and the results are shown in Figure 6 and Table S9. Generally, cell apoptosis was more prominent in treatments with higher concentrations of robenacoxib, especially 500 and 1000 μM, in a dose‐dependent manner, but without statistically significant differences (*p* > 0.05). However, comparing the results of different cells treated with similar drug concentration, the apoptotic rates of FISS‐12 cells at all concentrations were higher than those of FISS‐7 and FM cells.

## 4 DISCUSSION

Feline injection‐site sarcoma (FISS) is a highly invasive tumour associated with injective events in cats, but its tumorigenesis has not been studied clearly. Currently, there is a well‐known consensus that FISSs develop from chronic inflammation caused by injections. To investigate the association between tumorigenesis and chronic inflammation, cyclooxygenase‐2 (COX‐2), which is an important inflammation and tumorigenesis promotive factor that has been detected in FISSs in several studies, was selected as the target in this study. In the present study, the results demonstrated that robenacoxib, a highly selective COX‐2 inhibitor, has an inhibitory effect on cell viability, migration and colonigenesis; it promotes cell apoptosis in a dose‐dependent manner in FISS‐derived cells, suggesting that COX‐2 may play a role in FISS development, and it may have the potential to be used as adjuvant therapy in FISS, especially for interfering with migratory ability. However, these effects did not correlate with COX‐2 expression in FISS cells.

The results of ICC, IHC staining and western blotting showed that there was no significant difference in COX‐2 expression levels among these cells. Additionally, based on mRNA sequencing of these two FISS‐derived primary cells, there was no significant upregulation or downregulation of the COX‐2 gene when compared to that of the FM cells (data not shown). However, when comparing the effects of robenacoxib on cell viability, migration, colony formation and apoptosis, a dose‐dependent effect was observed in all the primary cells in each assay. It has been reported that preoperative administration of COX‐2 inhibitors could reduce the risks of metastasis in human medicine.^25^ Interestingly, when the concentration of robenacoxib was lower than its IC_50_, the FM cells demonstrated more active migration, but FISS primary cells showed relatively enhanced inhibitory effects, suggesting that FISS cells are more susceptible to robenacoxib in terms of migratory ability at relatively lower concentrations, such as 125 and 250 μM. Therefore, robenacoxib might induce a similar effect on FISSs, as it does on human cancers. In contrast, the results of the TUNEL assay demonstrated that FISS‐7 cells tended to be more resistant to the induction of apoptosis by robenacoxib than FISS‐12 and FM cells (*p* > 0.05), which is contrary to the IC_50_ results, indicating that robenacoxib might cause cell death via other mechanisms.

The source of COX‐2 in cultured cell lines cannot mimic the natural environment of COX‐2 expression during the inflammatory response in tissues and in vivo due to the lack of COX‐2‐expressing inflammatory cells. However, several studies have shown that epithelial and mesenchymal neoplastic cells can express COX‐2 as well.^28^ In canines, COX‐2 protein overexpression has been identified in mesenchymal and epithelial tumours.^44^, ^45^, ^46^, ^47^, ^48^, ^49^, ^50^, ^51^ Additionally, in previous studies, the expression of COX‐2 detected by immunohistochemistry varied among FISSs, but the positive rates of COX‐2 expression in the FISS‐affected cats ranged between 0%–97%.^30^, ^34^, ^35^, ^36^ It has also been reported that COX‐2 expression could be detected in neoplastic cell lines of canine transitional cell carcinoma and feline oral squamous cell carcinoma by immunohistochemical staining, suggesting that COX‐2 inhibitors could be potential candidates for anticancer agents directly affecting neoplastic cells in dogs and cats.^52^ In our study, the immunoreactivity of COX‐2 in FFPE tissues was both positive, but the positive cells were <5%, which might be affected by the preservation of the FFPE tissue blocks and consequently interfere with protein detection by antibodies. Because all the primary cells in our study expressed COX‐2, the dose‐dependent inhibitory effects, suggestive of a potential adjuvant therapy for FISSs, might vary in different cats affected by FISSs. However, our results also demonstrated that the expression level of COX‐2 could not be directly correlated with the susceptibility of FISS‐derived primary cells, further investigation is needed to elucidate the underlying mechanism of action of robenacoxib using more FISS primary cells with variable COX‐2 expression levels.

Although no published study has investigated the effect of robenacoxib or other COX inhibitors on FISS‐derived cells in vitro, the antitumor effects of robenacoxib have been investigated in canine primary cells derived from osteosarcoma and gastrointestinal stromal tumours.^53^ The sensitivities of COX‐2 inhibitors in all cell lines were not associated with the expression of COX‐2, and the inhibitory effects occurred only at relatively high concentrations, which is similar to our findings that the FISS‐7 cells were more susceptible to robenacoxib, despite the fact that FISS‐12 cells had relatively higher COX‐2 expression based on the quantitative results of western blotting, and the inhibitory responses were usually observed in treatments with 500 or 1000 μM of robenacoxib. This evidence indicates that the antitumor effects of robenacoxib and other COX inhibitors might be independent of COX‐2‐mediated pathways, which has also been reported in many studies in human medicine.^54^, ^55^ Additionally, a study evaluating the effect of the COX‐2 selective inhibitor celecoxib on canine tumour cells demonstrated similar findings.^56^ Upstream of COX‐2, NF‐κB is considered to be one of the important factors of COX‐2‐independent anti‐tumour effects and can also regulate the functions of cell apoptosis and inflammatory factor expression.^57^, ^58^ Cyclooxygenase‐2 selective inhibitors, such as etoricoxib, rofecoxib and lumiracoxib can inhibit the activation of the NF‐κB pathway to different degrees and can also change the expression of COX‐2 to varying levels.^59^, ^60^ In contrast, celecoxib can promote the apoptosis‐inducing effects of NF‐κB or other factors, such as phosphoinositide‐dependent kinase‐1 and its substrate pathways, leading to the induction of COX‐2‐independent apoptosis induction.^58^, ^60^ Celecoxib also inhibits angiogenesis by inhibiting the activation of Egr‐1.^60^ Thus, the mechanism of the COX‐2‐independent effects against tumours varies among different COX inhibitors. Robenacoxib is a relatively novel drug with limited studies on its antitumor effects. Further investigation is needed to elucidate the detailed mechanism and possible COX‐2 independent pathways and factors of robenacoxib.

In conclusion, robenacoxib generally exhibited a dose‐dependent inhibition in primary cells derived from FISSs and normal tissues at high concentrations, and FISS cells showed higher susceptibility to robenacoxib on cell migration. Although the mechanism of the anti‐tumour effects of robenacoxib and the role of COX‐2 in the tumorigenesis of FISS still require further studies, based on the findings in the present study, COX‐2 can be considered as a potential therapeutic target and COX‐2 selective inhibitors might be used as an adjuvant treatment for FISSs but further in vivo investigation is still needed.

## AUTHOR CONTRIBUTIONS

**Chen‐Hui Lu:** Data curation (lead); formal analysis (lead); investigation (lead); project administration (lead); writing – original draft (lead). **Shu‐Han Yu:** Methodology (supporting); resources (supporting). **Ching‐Ho Wu:** Methodology (equal); resources (equal). **Jason Lih‐Seng Yeh:** Methodology (supporting); resources (equal). **Hui‐Wen Chang:** Conceptualization (supporting); resources (supporting); supervision (supporting); validation (equal). **Chian‐Ren Jeng:** Supervision (supporting); validation (supporting). **Yen‐Chen Chang:** Conceptualization (lead); funding acquisition (lead); supervision (equal); validation (equal); writing – review and editing (lead).

## FUNDING INFORMATION

This work was supported by the National Science and Technology Council of Taiwan, Republic of China (NSTC 111‐2313‐B‐002‐058 to Y.‐C. Chang) and National Taiwan University, Taiwan, Republic of China (112L894803 to Y.‐C. Chang).

## CONFLICT OF INTEREST STATEMENT

The authors declare that they have no competing interests.

## Supporting information


Appendix S1

## Data Availability

The data in this study are available from the corresponding author upon reasonable request.
