# Supplementary material for: Effects of selective cyclooxygenase‐2 inhibitor robenacoxib on primary cells derived from feline injection‐site sarcoma
Source: J Cell Mol Med. 2023 Jun 19;27(15):2183–93. doi: 10.1111/jcmm.17717 (PMC10399534; doi:10.1111/jcmm.17717)
Supplement: Supplementary file 1 — Appendix S1 [file JCMM-27-2183-s001.docx]

**
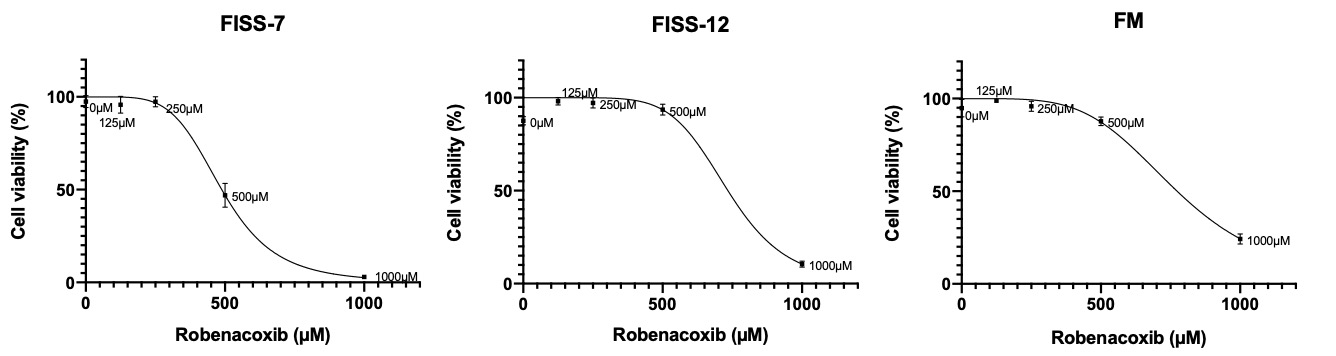
**

**Supplementary Figure 1.** Half maximal inhibitory concentration (IC_50_) curve of robenacoxib in FISS and FM primary cells at 72 h after treatment.

**
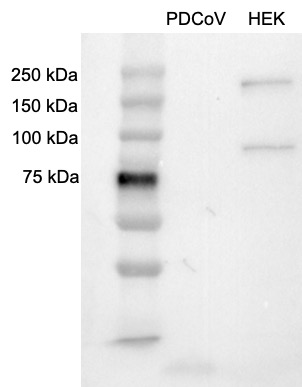
**

**Supplementary Figure 2.** The negative and positive controls of western blot using anti-COX-2 antibody. The positive and negative controls were the cell lysate from human embryonic kidney 293 cell line (HEK) and the recombinant protein of porcine deltacoronavirus spike protein (PDCoV), respectively. The COX-2 protein band is located between 75 and 100 kDa.

**Supplementary table 1. The signalments of the donor cats used in the primary cell isolation of feline injection-site sarcomas (FISS) and normal feline fibroblast (FM).**

| **Cell line** | **Breed** | **Sex** | **Age (year)** | **Location** | **Grade** |
| --- | --- | --- | --- | --- | --- |
| FISS-7 | Mixed | Mc | 5 | Right scapula | III |
| FISS-12 | Domestic short hair | Fsp | 9 | Left trunk | III |
| FM | Scottish fold | Mc | 12 | Trunk | - |

Mc, male castrated; Fsp female spayed.

**Supplementary table 2. The effect of the cell proliferation of FISS-7 of incubation at different concentrations of robenacoxib on 24 and 72 hours for calculating half-maximal inhibitory concentration (IC50).**

| Robenacoxib (µM) | 24 hours (%) | 72 hours (%) |
| --- | --- | --- |
| Control | 100.0 ± 10.9 | 100.0 ± 6.5 |
| 0 | 99.7 ± 4.4 | 97.4 ± 3.2 |
| 125 | 90.9 ± 3.5 | 95.8 ± 4.5 |
| 250 | 79.6 ± 16.8 | 97.3 ± 2.7 |
| 500 | 43.7 ± 3.9 | 47.0 ± 6.4 |
| 1000 | 22.7 ± 3.5 | 3.0 ± 0.6 |

The results are expressed as the mean percentage of control with standard error of mean. Group of concentration 0 µM means the cells were cultured in drug-free and DMSO containing medium. The cells in control group were cultured in drug and DMSO-free medium.

**Supplementary table 3. The effect of the cell proliferation of FISS-12 of incubation at different concentrations of robenacoxib on 24 and 72 hours for calculating half-maximal inhibitory concentration (IC50).**

| Robenacoxib (µM) | 24 hours (%) | 72 hours (%) |
| --- | --- | --- |
| Control | 100.0 ± 6.4 | 100.000 ± 1.5 |
| 0 | 79.0 ± 16.7 | 87.518 ± 2.3 |
| 125 | 102.1 ± 15.3 | 98.006 ± 2.1 |
| 250 | 99.7 ± 7.9 | 97.147 ± 2.7 |
| 500 | 98.4 ± 3.3 | 93.510 ± 3.0 |
| 1000 | 52.0 ± 5.4 | 10.492 ± 1.7 |

The results are expressed as the mean percentage of control with standard error of mean. Group of concentration 0 µM means the cells were cultured in drug-free and DMSO containing medium. The cells in control group were cultured in drug and DMSO-free medium.

**Supplementary table 4. The effect of the cell proliferation of FM cells of incubation at different concentrations of robenacoxib on 24 and 72 hours for calculating half-maximal inhibitory concentration (IC50).**

| Robenacoxib (µM) | 24 hours | 72 hours |
| --- | --- | --- |
| Control | 100.0 ± 0.8 | 100.0 ± 4.9 |
| 0 | 73.2 ± 16.0 | 94.8 ± 4.7 |
| 125 | 92.5 ± 3.5 | 98.9 ± 0.6 |
| 250 | 95.1 ± 4.3 | 95.9 ± 2.6 |
| 500 | 91.3 ± 3.9 | 87.7 ± 2.3 |
| 1000 | 47.5 ± 7.3 | 24.3 ± 2.7 |

The results are expressed as the mean percentage of control with standard error of mean. Group of concentration 0 µM means the cells were cultured in drug-free and DMSO containing medium. The cells in control group were cultured in drug and DMSO-free medium.

**Supplementary table 5. The effect on the cell migration of FISS-7 primary cells under different concentrations of robenacoxib following 24 hours of incubation at different concentrations evaluated by wound healing assay.**

| Robenacoxib (µM) | Average width of T0 (µm) | Mean ratio of width T1/T0 (%) | *p*-value |
| --- | --- | --- | --- |
| Control | 74.4 | 56.1 ± 8.7 |  |
| 0 | 122.9 | 74.5 ± 9.4 | 0.7281 |
| 125 | 119.0 | 36.8 ± 20.6 | 0.6921 |
| 250 | 166.9 | 74.5 ± 20.1 | 0.7297 |
| 500 | 254.0 | 90.3 ± 15.5 | 0.2099 |
| 1000 | - | - | - |

-: Not available because the cells were sloughed off and the wound was not recognizable. T0 represents the timepoint after the wound was made. T1 represents the timepoint of T0 after 24 hours. The *p-value* is the probability of observing treatment effect compared to the control group.

**Supplementary table 6. The effect on the cell migration of FISS-12 primary cells under different concentrations of robenacoxib following 24 hours of incubation at different concentrations evaluated by wound healing assay.**

| Robenacoxib (µM) | Average width (µm) | Mean ratio of width T1/T0 (%) | *p*-value |
| --- | --- | --- | --- |
| Control | 189.8 | 55.7 ± 4.2 |  |
| 0 | 239.5 | 60.3 ± 9.9 | 0.9943 |
| 125 | 121.0 | 35.9 ± 14.0 | 0.2712 |
| 250 | 155.2 | 35.6 ± 5.8 | 0.2587 |
| 500 | 279.3 | 55.8 ± 11.1 | 1.0000 |
| 1000 | 299.6 | 66.6 ± 3.6 | 0.8132 |

T0 represents the timepoint after the wound was made. T1 represents the timepoint of T0 after 24 hours. The *p-value* is the probability of observing treatment effect compared to the control group.

**Supplementary table 7. The effect on the cell migration of FM primary cells under different concentrations of robenacoxib following 24 hours of incubation at different concentrations evaluated by wound healing assay.**

| Robenacoxib (µM) | Average width (µm) | Mean ratio of width T1/T0 (%) | *p*-value |
| --- | --- | --- | --- |
| Control | 0 | 0 |  |
| 0 | 0 | 0 | 1.000 |
| 125 | 0 | 0 | 1.000 |
| 250 | 0 | 0 | 1.000 |
| 500 | 179.7 | 53.6 ± 12.4* | <.0001 |
| 1000 | 243.8 | 72.8 ± 7.2* | <.0001 |

T0 represents the timepoint after the wound was made. T1 represents the timepoint of T0 after 24 hours. The *p-value* is the probability of observing treatment effect compared to the control group. * indicates statistical difference when compared to the control group (*p*<0.05).

**Supplementary table 8. The inhibitory effect of robencoxib on colony formation in the primary cells following 2 week incubation at different concentrations of robenacoxib.**

|  |  |  | Numbers of colonies | | |  |
| --- | --- | --- | --- | --- | --- | --- |
| Robenacoxib (µM) | FISS-7 | *p*-value | FISS-12 | *p*-value | FM | *p*-value |
| Control | 27. 7 ± 1.2 |  | 66.0 ± 21.8 |  | 19.0 ± 7.8 |  |
| 0 | 23.0 ± 4.4 | 0.8450 | 69.0 ± 5.3 | 0.9997 | 20.3 ± 2.3 | 0.9994 |
| 125 | 25.0 ± 1.7 | 0.9831 | 49.0 ± 1.7 | 0.6110 | 17.3 ± 3.1 | 0.9983 |
| 250 | 27.7 ± 2.1 | 1.0000 | 47.0 ± 14.2 | 0.5014 | 15.3 ± 5.0 | 0.9433 |
| 500 | 7.3 ± 8.5* | 0.0022 | 0.3 ± 0.6* | 0.0004 | 2.3 ± 2.3* | 0.0131 |
| 1000 | 0* | 0.0001 | 0* | 0.0004 | 0* | 0.0049 |

The *p-value* is the probability of observing treatment effect compared to the control group. * represents statistically significance when compared to the control group (*p*<0.05).

**Supplementary table 9. The cell apoptotic rate in the primary cells following incubation at different concentrations of robenacoxib for 24 hours detected by TUNEL assay.**

|  |  | |  | Apoptotic rate (%, apoptosis / total cell count) | | | |  |
| --- | --- | --- | --- | --- | --- | --- | --- | --- |
| Robenacoxib (µM) | FISS-7 | *p*-value | | | FISS-12 | *p*-value | FM | *p*-value |
| Control | 0.4 ± 0.3 |  | | | 0.0 ± 0.0 |  | 0.1 ± 0.1 |  |
| 0 | 0.7 ± 0.7 | 1.0000 | | | 0.1 ± 0.1 | 1.0000 | 0.1 ± 0.1 | 1.0000 |
| 125 | 0.4 ± 0.3 | 1.0000 | | | 0.8 ± 0.9 | 1.0000 | 1.8 ± 0.6 | 0.9982 |
| 250 | 0.3 ± 0.1 | 1.0000 | | | 2.8± 4.6 | 0.9965 | 1.2 ± 1.3 | 0.9997 |
| 500 | 2.2 ± 3.0 | 0.9168 | | | 12.1 ± 11.9 | 0.3547 | 4.8 ± 5.9 | 0.8539 |
| 1000 | 3.0 ± 3.3 | 0.6980 | | | 18.8 ± 7.2 | 0.0579 | 12.0 ± 8.6 | 0.0743 |

The *p-value* is the probability of observing treatment effect compared to the control group.
